# Male Sexual Dysfunction-Related Medical Comorbidities in a Tertiary Care Center, Western Region, Saudi Arabia

**DOI:** 10.7759/cureus.41732

**Published:** 2023-07-11

**Authors:** Mohammed Bogari, Basil A Alzahrani, Afnan S Aghashami, Abdullah Mady S Alsubeay, Fadil Hassan, Mohamed E Ahmed, Mohammed M Almuqati

**Affiliations:** 1 College of Medicine, King Saud Bin Abdulaziz University for Health Sciences, Jeddah, SAU; 2 Urology, King Fahad Armed Forces Hospital, Jeddah, SAU; 3 Urology, King Abdullah International Medical Research Center, Jeddah, SAU; 4 Basic Sciences, King Saud Bin Abdulaziz University for Health Sciences, Jeddah, SAU; 5 Urology, King Abdullah Medical City, Jeddah, SAU

**Keywords:** ischemic heart disease, diabetes, saudi arabia, erectile dysfunction, male sexual dysfunction

## Abstract

Introduction: Sexual functionality is considered a vital component of human life and quality of life. Issues with sexual functionality can be a source of distress, lower self-esteem, and lower quality of life. Early detection of medical comorbidities can significantly lower the effect on sexual function. In Saudi Arabia, studies investigating the association between medical comorbidities and male sexual dysfunction (MSD) are limited. Therefore, our goal was to fill this knowledge gap.

Aim: This study aimed to analyze and elaborate on all cases of MSD at a tertiary hospital, in Jeddah, Saudi Arabia from 2016 to 2021.

Method: This is a cross-sectional retrospective study. The medical records of 321 patients diagnosed with MSD from 2016 to 2021 were reviewed retrospectively. The age, sex, type of sexual dysfunction, comorbidities, and lipids profile were some of the factors obtained from the patient’s computerized medical records.

Results: The study population included 321 men with MSD and a mean age of approximately 53 years (SD=11.5). Among the sexual dysfunction pattern, only erectile dysfunction (ED) was found in 279 (86.9%) patients. ED duration lasted one to five years in 169 (52.8%) patients. Most of the patients (196, 61.1%) had mild ED severity. Medical causes were seen in 278 (80.4%) patients. The most frequent comorbidities were diabetes mellitus (DM) in 179 (55.8%) patients, hypertension (HTN) in 155 (48.2%) patients, and dyslipidemia in 113 (35.2%) patients. Smoking was not a risk factor for ED. The risk of having a severe form of ED was associated with idiopathic causes, HTN, DM, and ischemic heart disease (IHD). The risk of having a long duration of ED was related to idiopathic causes of ED and high serum creatinine levels.

Conclusion: In conclusion, patients diagnosed with DM, HTN, and IHD are at greater risk to experience a severe form of ED. It is crucial to keep erection function in mind for patients with DM, HTN, and IHD as this is associated with severe ED.

## Introduction

Sexual functionality is considered a vital component of human life and quality of life. Issues with sexual functionality can be a source of distress, lower self-esteem, and lower quality of life. It also can cause issues in serious relationships (REF) [1].

Based on studies in the USA, it appears that sexual dysfunctions are very common in both sexes, ranging from 10% to 52% of males [2]. The prevalence of ED in Asia varies between 9% and 73% [3]. According to research by the National Health and Social Life Survey, out of 1749 women, 43% reported having problems with their sexual health [4]. For premature ejaculation, a study conducted in China found that the prevalence rate for adults aged 40-80 years old was 19.5% [5]. 

Male sexual dysfunction (MSD) is characterized by a change in at least one of the essential sexual functions (desire, erection, orgasm, and ejaculation). MSD may also cause discomfort during sexual activities as well as unhappiness with one's sexual life [6]. Human sexual function is highly complex and includes both physiologic and psychological elements. Because the diagnosis is dependent on clinical evidence, it is essential to have a thorough sexual history and a focused physical examination [7].

Men's sexual dysfunction can have a variety of reasons, each with a unique set of risk factors and therapies. A lack of interest in thinking about or engaging in sexual activity, whether alone or with a partner, is a sign of low sexual desire. The persistent or recurrent inability to achieve or sustain a penile erection strong enough to provide sexual satisfaction is known as erectile dysfunction (ED) [8].

The contributing factors of MSD are addressed depending on the biopsychosocial model [9]. The biological factors include age with an association with the underlying health/comorbidities. Some studies showed a strong relationship between diabetes millets (DM) and ED with an incidence of 35% to 90%, especially in poorly controlled DM [10]. Other conditions common in men with DM are obesity, hypertension (HTN), hyperlipidemia, smoking, cardiovascular diseases, and urinary tract diseases; moreover, they are independent risk factors for sexual dysfunction [11]. In addition, psychological disorders, such as depression and anxiety, were found to have a significant impact on ED/level of desire. Also, some of the medications used to treat psychiatric conditions and drug abuse have a clear association with sexual dysfunction [11].

Worldwide, many studies discussed the association between medical comorbidities and MSDs. However, in Arab society, and specifically in Saudi Arabia, this topic is still highly sensitive. Researchers must break the silence about sexual health in Saudi Arabia. Hence, this study aimed to analyze and elaborate on all cases of MSD at a tertiary hospital, in Jeddah, Saudi Arabia over the past five years.

## Materials and methods

Sampling and participants

For this study, a convenience sampling technique was used as the primary target participants for patients presenting with ED. The inclusion criteria for participants in the study were any patient that was presented with ED and was over the age of 18 years. However, any patient who does not have an androgen profile will be excluded.

The patients’ data were collected from electronic medical records, BestCare. In total, 321 patient records presenting with ED were found during the five-year period of 2016-2021. The demographic data, medical comorbidities, and laboratory results, such as creatinine level (umol/L), HgA1c, total testosterone (mmol/L), prolactin (ug/L), LH (IU/L), FSH (IU/L), TSH, LDL (mmol/L), total cholesterol (mmol/L), and triglyceride (mmol/L), were collected.

The study took place at King Abdulaziz Medical City (KAMC), Jeddah, Saudi Arabia, and the ethical approval for the study was obtained from the Institutional Review Board of King Abdullah International Medical Research Center (approval number: NRJ22J/246/10).

Statistical analysis

Data collected was stored, prepared, and coded in Excel sheets prior to the data analysis process. JMP version 15 software was used to analyze the data. Categorical variables were presented in frequencies and percentages and quantitative continuous variables were described by measures of descriptive statistics including means and SD or median and IQR based on normality assessment. A significant association between the categorical variables was examined using the Chi-square test for association, and ANOVA Kruskal Wallis tests were conducted to test significant differences in continuous variables. A p-value of less than or equal to 0.05 was considered significant.

## Results

The study population included a total of 321 men eligible after excluding subjects who had incomplete data or were not diagnosed with ED. The characteristics of the participants are listed in (Table 1). Their mean age was approximately 53 years (SD=11.5) with a mean BMI of 29.7 kg/m^2^ (SD=3.2). The marital status was available for all patients, of which 293 men (91.3%) were married to one wife while only 28 (8.7%) have more than one. The ED patterns were categorized into ED only, which was seen in 279 (86.9%) patients, ED with premature ejaculation (PE), reported by 27 (8.4%) patients, and other patterns, which include ED + premature ejaculation + low sexual desire seen in 15 (4.7%) of the participants. The ED duration was less than one year in 90 men (28.1%), the majority 169 men (52.85) had ED for one to five years, 48 men (15%) were more than five years, and 13 (4.1%) were more than ten years. In the outpatient clinic, the ED severity was reported as mild ED by 196 men (61.1%), moderate ED by 110 (34.3%), and severe ED by 15 (4.6%) (Table 1).

**Table 1:** Basic characteristics of participants BMI: body mass index; ED: erectile dysfunction; NA: not applicable

Variable	N=321
Age	53.1±11.5
BMI	29.7±5.6
Number of wives	
One	293 (91.3)
More than one	28 (8.7)
Sexual dysfunction pattern	
ED only	279 (86.9)
ED with premature ejaculation	27 (8.4)
Others	15 (4.7)
ED duration	
< 1 year	90 (28.1)
Years	169 (52.8)
>5 years	48 (15.0)
>10 years	13 (4.1)
ED severity	
Mild	196 (61.1)
Moderate	110 (34.3)
Severe	15 (4.6)
ED etiology	
Organic	258 (80.4)
Psychological	33 (10.3)
Mixed	18 (5.6)
Idiopathic	12 (3.7)
Organic cause	
Arthritogenic	267 (83.4)
Neurogenic	10 (3.1)
NA	43 (13.5)
Smoking	
No	233 (72.6)
Current smoker	61 (19.0)
Ex-smoker	27 (8.4)

In 321 patients, the most reported ED etiology was organic (80.4%), followed by psychological (10.3%), mixed (5.6%), and idiopathic (3.7%) causes. In addition, the organic causes were grouped into arteriogenic ED, which is caused by insufficient arterial blood supply to the cavernous bodies, and neurogenic ED. A total of 267 (83.4%) men had arteriogenic ED and ten (3.1%) men had neurogenic ED. A total of 43 (13.5%) men were categorized as not applicable and had a psychological or idiopathic cause (Table 1).

In all, there were 61 smokers (19%), 27 ex-smokers (8.4%), and the majority 233 men (72.6%) were non-smokers (Table 1).

Seven major comorbidities were identified among the 321 patients with ED. DM was the most frequent comorbidity with a greater prevalence (55.8%) and median HgA1c (6.9%), followed by HTN (48.2%), and dyslipidemia (35.2%). Liver (4.4%) and kidney (8.4%) diseases were the least associated with MSD in this study (Table 2). 

**Table 2:** Comorbidities HTN, hypertension; IHD, ischemic heart disease

Comorbidity	N=321 (%)
HTN	155 (48.2)
Dyslipidemia	113 (35.2)
IHD	62 (19.3)
Cerebrovascular	14 (4.4)
Liver disease	8 (2.5)
Kidney disease	27 (8.4)
Diabetes	179 (55.8)

A few laboratory tests were performed on the sample, including creatinine level, HgA1c, total testosterone, prolactin, and others as presented in (Table 3). The median level of creatinine was 78 (IQR: 19.3), the HgA1c median level was 6.9 (IQR: 3.1), and the median prolactin level was 8 (IQR: 5.9) (Table 3).

**Table 3:** Patient laboratory results HgA1C, hemoglobin A1C; LH, luteinizing hormone; FSH, follicle-stimulating hormone; TSH, thyroid stimulating hormone; LDL, low-density lipoprotein

Parameter	Median (IQR)
Creatinine level	78 (19.3)
HgA1c	6.9 (3.1)
Total testosterone	14.8 (9.0)
Prolactin	8 (5.9)
LH	3.5 (3.0)
FSH	3.5 (3.3)
TSH	1.9 (1.6)
LDL	2.6 (1.3)
Total cholesterol	4.6 (1.6)
Triglyceride	1.6 (1.2)

In Table 4, the association between ED etiologies and medical comorbidities and ED severity was illustrated. There was a significant association between organic, psychological, mixed, and idiopathic causes in relation to the ED severity with a p-value of <0.0001. Among all causes of ED, most of the patients with organic cause (68%), which represent most of the participants n=258, reported mild ED presentation while those who reported moderate ED were likely found to have psychological (57%), mixed (66.67%), or idiopathic (83.33%) causes. Severe ED was significantly associated with idiopathic causes (8.33%). In addition, this study revealed no significant association between ED severity and smoking with a p-value of <0.5815 (Table 4).

**Table 4:** Association between ED etiology, medical comorbidities, and ED severity *P value is significant. HTN, hypertension; IHD, ischemic heart disease; DM, diabetes mellitus

	Erectile dysfunction severity			
Variable	Mild, N=19 (%)	Moderate, N=110 (%)	Severe, N=15 (%)	P-value
Cause				0.0001*
Organic	77 (68)	69 (26.74)	12 (4.65)	
Psychological	13 (39.39)	19 (57.58)	1 (3.03)	
Mix	5 (27.27)	12 (66.67)	1 (5.56)	
Idiopathic	1 (8.33)	10 (83.33)	1 (8.33)	
Smoking				0.5815
No	146 (62.66)	78 (33.38)	9 (3.86)	
Current	33 (54.10)	23 (37.70)	5 (8.20)	
Ex	17 (62.96)	9 (33.33)	1 (3.70)	
HTN				0.0052*
Yes	102 (65.80)	43 (27.67)	10 (6.45)	
No	94 (56.62)	67 (40.36)	5 (3.01)	
Dyslipidemia				0.0564
Yes	79 (69.91)	30 (27.55)	4 (3.54)	
No	117 (56.25)	80 (38.46)	11(5.29)	
IHD				0.0443*
Yes	39 (62.9)	10 (16.12)	13 (20.96)	
No	157 (60.6)	100 (38.6)	2 (0.77)	
Cerebrovascular				0.1351
Yes	6 (42.86)	6 (42.86)	2 (14.29)	
No	190 (61.89)	104 (33.88)	13 (4.23)	
Liver disease				0.2190
Yes	3 (37.50)	5 (62.50)	0 (0.00)	
No	193 (61.66)	105 (33.55)	15 (4.79)	
Kidney disease				0.1319
Yes	12 (44.44)	14 (51.85)	1 (3.70)	
No	184 (62.59)	96 (32.65)	14 (4.76)	
DM				0.0011*
Yes	126 (70.4)	43 (24.0)	10 (5.6)	
N0	70 (49.3)	67 (47.2)	5 (3.5)	

Furthermore, the study showed a significantly strong association between ED severity and HTN (p-value: <0.00528). 65.8% of men with HTN reported mild ED presentation, 27.67% were found to have moderate ED, and 6.45% reported severe ED. On the other hand, 94 (56.62%) patients with no history of HTN reported mild ED, 67 (40.36%) had moderate ED, and 5 (3.01) had severe ED.

A comparison of ED severity in patients with ischemic heart disease (IHD) and patients without IHD is summarized in Table 4, about 62.9% of IHD patients reported mild ED, 16.12% had moderate ED, and 20.96% had severe ED. There was a significant association between IHD and ED severity with a p-value of <0.0443. In addition, patients who had DM as a comorbidity showed a significant association with the severity of ED (P-value of <0.001). 70.4% of men with DM reported mild ED presentation, 24% were found to have moderate ED, and 10% reported severe ED.

The association between reported yes dyslipidemia vs no dyslipidemia and ED severity was high but did not reach statistical significance (69.91% vs 56.25%, p-value: 0.0564). Likewise, there was no significant association between other identified comorbidities and ED severity.

The association between ED etiology, medical comorbidity, and ED duration is illustrated in Table 5. There was a significant association between one and five years of ED duration and ED etiologies including organic cause (n=137, 53.1%), psychological cause (n=19, 57.58%), mixed (n=8, 44.44%), and idiopathic cause (n=5, 41.67%, p-value: <0.02).

**Table 5:** Association between ED etiology, medical comorbidities, and ED duration *P-value is significant. HTN, hypertension; IHD, ischemic heart disease; DM, diabetes mellitus

Variable	Less than 1 year, N=90 (%)	1-5 years, N=169 (%)	More than 5 years N=48, (%)	More than 10 years N=13, (%)	P-value
Cause					0.0275*
Idiopathic	1 (8.33)	5 (41.67)	3 (25.00)	3 (26.00)	
Organic	76 (29.46)	137 (53.10)	38 (14.73)	7 (2.71)	
Psychological	7 (21.21)	19 (57.58)	5 (15.15)	2 (6.06)	
Mix	7 (38.88)	8 (44.44)	2 (11.11)	1 (5.55)	
Smoking					0.37
Ex	3 (11.11)	17 (62.96)	5 (18.52)	2 (7.41)	
No	70 (30.04)	117 (50.21)	37 (15.88)	9 (3.86)	
Current	18 (29.50)	35 (57.37)	6 (9.83)	2 (3.27)	
HTN					0.8184
Yes	42 (28.00)	78 (52)	25 (16.67)	10 (3.33)	
No	48 (28.91)	91 (54.81)	23 (13.85)	4 (2.40)	
Dyslipidemia					0.4688
Yes	32 (28.32)	57 (50.44)	21 (18.58)	3 (2.65)	
No	58 (28.02)	112 (54.11)	27 (13.04)	10 (4.83)	
IHD					0.5153
Yes	11 (17.7)	30 (48.38)	9 (14.51)	12 (19.3)	
No	79 (30.52)	139 (53.66)	39 (15.05)	2 (0.77)	
Cerebrovascular					0.6350
Yes	2 (14.29)	9 (64.29)	2 (14.29)	1 (7.14)	
No	88 (28.76)	160 (52.29)	46 (15.03)	12 (3.92)	
Liver disease					0.4386
Yes	1 (12.50)	4 (50)	2 (25.00)	1 (12.50)	
No	89 (28.53)	165 (52.88)	46 (14.74)	12 (3.85)	
Kidney disease					0.5590
Yes	5 (18.52)	15 (55.56)	5 (18.52)	2 (7.41)	
No	85 (29.01)	154 (52.56)	43 (14.68)	11 (3.75)	
DM					0.1511
Yes	46 (25.8)	98 (55.1)	30 (16.9)	4 (2.3)	
No	44 (31.0)	71 (50.0)	18 (12.7)	9 (6.4)	

Moreover, Table 5 showed no significant association between ED duration and smoking, dyslipidemia, and other comorbidities.

The association between lab parameters/values and ED severity is identified in Table 6. The prolactin level significantly increased in severe ED (28.90±3.91, p-value: <0.0001) while patients with mild and moderate ED showed normal prolactin levels (10.10±1.18 vs 9.86±1.33, respectively). However, the BMI, HgA1c, creatinine, total testosterone, TSH, LH, and FSH did not show any clinical significance or association with ED severity.

**Table 6:** Association between lab value and ED severity *p-value is significant. BMI, body mass index; HgA1C, hemoglobin A1C; LH, luteinizing hormone; FSH, follicle-stimulating hormone; TSH, thyroid stimulating hormone; LDL, low-density lipoprotein

Count	Mild	Moderate	Severe	P value
BMI (Mean SD)	29.33±0.40	30.43±0.53	28.55±1.45	0.1917
Age (Mean SD)	53.8±11.4	51.9±11.7	52.4±11.4	0.1922
HgA1c (Mean SD)	7.44±0.14	7.03±0.19	7.20±0.50	0.2296
Creatinine (Mean SD)	87.03±7.62	102.15±10.04	83.26±26.18	0.4503
Total testosterone (Mean SD)	15.76±0.66	16.86±0.87	18.28±2.47	0.4273
Prolactin (Mean SD)	10.10±1.18	9.68±1.33	28.90±3.91	0.0001*
TSH (Mean SD)	2.89±0.39	1.96±0.57	2.17±1.46	0.3961
LH (Mean SD)	4.21±0.35	4.61±0.44	5.05±1.05	0.7293
FSH (Mean SD)	4.65±0.55	4.93±0.68	4.93±1.06	0.9466

Table 7 demonstrated the association between different lab parameters and ED duration. Creatinine level was significantly high in patients with ED for more than ten years (184.5±28.9, p-value: <0.0094).

**Table 7:** Association between lab values and ED duration *p-value is significant. BMI, body mass index; HgA1C, hemoglobin A1C; LH, luteinizing hormone; FSH, follicle-stimulating hormone; TSH, thyroid stimulating hormone; LDL, low-density lipoprotein

Count	Less than 1 year	1-5 years	More than 5 years	More than 10 years	P-value
BMI (Mean±SD)	29.42±0.95	29.85±0.43	27.8±1.56	30.11±0.81	0.5760
Age (Mean±SD)	50.9±11.9	53.7±11.4	54.4±10.4	54.4±12.3	0.5761
HgA1c (Mean±SD)	7.18±0.21	7.63±0.51	7.47±0.28	6.5±0.56	0.4251
Creatinine (Mean±SD)	82.46±10.7	93.41±7.81	80.58±14.45	184.5±28.9	0.0094*
Total testosterone (Mean±SD)	17.22±0.95	15.91±0.70	17.08±1.32	19.93±2.88	0.5173
Prolactin (Mean±SD)	9.76±1.74	10.94±1.28	12.69±2.37	9.84±4.48	0.7906
TSH (Mean±SD)	3.30±0.59	2.35±0.43	2.03±0.83	2.05±1.60	0.5125
LH (Mean±SD)	3.56±0.05	4.37±0.38	4.91±0.57	3.57±1.82	0.3395
FSH (Mean±SD)	4.26±0.78	4.47±0.58	6.17±3.39	0.88±3.41	0.3409

## Discussion

In the present study, 321 patients met the inclusion criteria, and the majority of this study patients had an age distribution of more than 60 years with a mean age of 53.1±11.5. In contrast to this study finding, Al Helali et al. showed an age distribution of fewer than 50 years with a mean age of 43.23±12.56 [12]. A different study done in Poland showed that patients aged 41-50 years had a higher prevalence of ED compared with patients aged below 40 [13]. 53% of those who were diagnosed with ED by Rösing et al. were above 70 years of age [14]. All these studies agreed that it is rare to be diagnosed with ED before the onset of 40 years. Although this study shows that as the person gets old the chance of having ED increases, there is no evidence between an increase in age and an increase in ED severity or duration. On the other hand, Shiri et al. showed that severe ED increased by 18% for each one-year age increment [15]. The differences in the findings can be explained by the fact that their study included both urban and rural areas. People who live in rural areas may not seek help and may not be compliant with treatment. Also, comorbidities and etiologies may explain why they had such findings.

ED (86.9%) was the most common type of MSD in the present study. Different studies also demonstrated that the most common form of sexual dysfunction is ED and PE [16,17]. Etiologies of ED were divided into organic, psychological, mixed, and idiopathic. In the present study, most patients with ED had organic causes (80.4%) followed by psychological causes (10.3%), mixed (5.6%), and idiopathic (3.7%), in contrast to Polland et al., which found that ED patients had a mental health cause more than organic. The majority of Polland et al. had depression (35.4%) and other mental health conditions (35.9%) as a cause of ED [18]. However, in our study, organic causes like DM (55.8%), HTN (48.2%), dyslipidemia (35.2%), and IHD (19.3%) were most commonly reported. Diabetes is highly prevalent in Saudi society and is a serious clinical and public health issue. One study reveals that DM prevalence in Saudi Arabia reached 30% [19]. This fact may explain why our study had DM as the most common medical comorbidity associated with ED. In this study, psychological causes of ED were seen mainly among patients diagnosed with depression and anxiety. This can be explained by the fact that both groups used selective serotonin reuptake inhibitors (SSRI), which lead to sexual dysfunction. 3.7% of this study patients had idiopathic causes of ED, and in those patients, there was no evidence of organic or psychologic cause. In addition, those patients were significantly associated with severe ED (8.33%) and were associated with a longer duration of ED (26%).

This study showed that 61.1% of the patients had mild ED, 34.3% had moderate ED, and 4.6% had severe ED. Another study showed that 20.2% of the patients had mild ED whereas 13.8% had moderate ED and 7.5% of men reported severe ED [20]. Furthermore, Shiri et al. showed a prevalence of severe ED of about 29% [15]. A different study reported that 48% of the population had severe ED [21]. ED severity levels vary greatly between different studies and this broad variation is attributable to differences in population size, age, medical comorbidities, and the fact that these studies were conducted at different times. In this study, DM, HTN, and IHD were the medical comorbidities that were significantly associated with severe ED. Our study found that 5.6% of patients with DM suffered from severe ED. However, there was no association between DM and ED duration. One systematic review also found a similar finding to our study, a high percentage of DM (53.3%) among ED patients [22]. Furthermore, patients who had IHD and/or HTN as comorbidities were associated with ED and were more likely to have a severe form of ED. 20,96% of patients with IHD had severe ED, and 6.45% with HTN developed severe ED. According to Kongkanand et al., 62.4% of HTN patients suffer from mild to severe degrees of ED, and males with heart disease constitute 64.2% of ED patients [23]. Additionally, studies have indicated that people with HTN and IHD experience more severe ED than the general population [22,24].

Most of this study patients (53.8%) had ED duration between one and five years. El-Sakka et al. also reported that the most common duration of ED is between one and five years with 59% [22]. Moreover, another study revealed that about one-half of their patients (48%) suffered from ED for a period of 1 to five years [13]. All these studies are local studies done in Saudi Arabia; this may explain why we had a similar result. These results suggest that patients with ED are more likely to recover within five years. However, the duration of ED can range from months to years. Depending on the underlying reason, you could live a lifetime with ED.

The mean BMI of this study was 29.7+11.5, which is considered high. A further study done by Liu and colleagues had a mean BMI of 24.7 kg [25]. Fillo et al. showed that the incidence rate of ED was directly attributed to the growing prevalence of obesity [26]. Obesity is a well-known risk factor for ED; however, the association between obesity and ED severity and duration has not been linked. Although this study showed that most patients with ED had high BMI, there was no evidence that patients with high BMI will result in severe ED or a long duration of ED. A cross-sectional study done by Liu et al. concluded that there is still no apparent connection between the various BMI classifications and the degrees of ED severity [25].

Smoking, in this study, was not highly prevalent among ED patients, only 19% were smokers. In addition to that, smoking was not associated with severe ED nor with a long duration of ED. A different study done in the same country revealed that smoking was not a risk factor for ED. This finding may reveal that smoking is not common in Saudi Arabia and that is why it was not associated with ED [27]. In contrast, a sample of 178 patients who were referred for an examination of impotence revealed a higher rate of cigarette smoking, with 58% of current smokers and 81% of current and ex-smokers combined [28].

In this study the most common lab value that was associated with severity of ED was prolactin. Patients with high prolactin levels mean (28.90) experienced a more severe form of ED. Previous studies also showed that high prolactin had a detrimental impact on erectile function [29]. In this study, patients with high serum creatinine were more likely to suffer a longer duration of ED. Serum creatinine with a mean of 184.5 experienced ED for more than 10 years. In other words, although CKD was not associated with the severity of ED, it was associated with a longer duration of ED. Many studies discussed the prevalence of ED and CKD, and it is estimated that almost 70% of men with CKD will report having ED at some point in their life [30]. Although the association between CKD and ED has been established, we need more studies about CKD and its effect on the duration of ED.

Limitations

Despite the promising results of the study, there are some limitations. First, the results of this study only apply within the Saudi context due to the different cultural and socio-demographic factors that differentiate it from Western countries. Second, this study includes a convenience sample of patients that presented with ED. This meant that the association between ED and some demographic factors such as smoking was not adequately captured. To understand this association better, a random sampling approach may provide better results. Finally, due to the use of patient records for data purposes, information on patients was limited to what was available on record.

## Conclusions

In conclusion, in the current study, the most common form of MSD is ED. Patients who were older than 50 years were more likely to have ED. Organic causes reported in this study were more than psychological causes. The most common medical comorbidity was DM, and there was a significant association between DM, HTN, IHD, and prolactin levels and severe forms of ED. However, there was no association between medical comorbidity and ED duration. The leading indicator for the long duration of ED was CKD. In this study, smoking was not statistically significant. It is essential to pay clinical attention to erection function in patients with DM, HTN, and IHD because they are the most seen in severe forms of ED.
